# Polydopamine-Coated Polymer Nanofibers for In Situ Protein Loading and Controlled Release

**DOI:** 10.1021/acsomega.4c00263

**Published:** 2024-03-13

**Authors:** Meina Zhang, Romy A. Dop, Haifei Zhang

**Affiliations:** † Department of Chemistry, University of Liverpool, Crown Street, Liverpool L69 7ZD, U.K.; ‡ Department of Clinical Infection, Microbiology and Immunology, Institute of Infection, Veterinary and Ecological Sciences, University of Liverpool, Liverpool L69 7ZD, U.K.

## Abstract

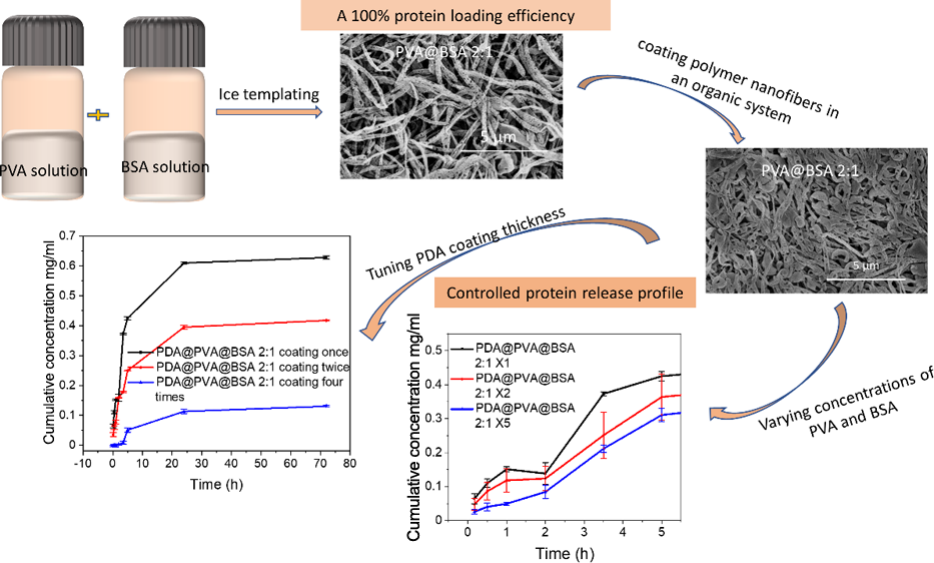

Nanofibrous polymeric materials, combined with protein therapeutics, play a significant role in biomedical and pharmaceutical applications. However, the upload of proteins into nanofibers with a high yield and controlled release has been a challenging issue. Here, we report the in situ loading of a model protein (bovine serum albumin) into hydrophilic poly(vinyl alcohol) nanofibers via ice-templating, with a 100% protein drug loading efficiency. These protein-loaded nanofibers were further coated by polydopamine in order to improve the nanofiber stability and achieve a controlled protein release. The mass ratio between poly(vinyl alcohol) and bovine serum albumin influenced the percentage of proteins in composite nanofibers and fiber morphology. More particles and less nanofibers were formed with an increasing percentage of bovine serum albumin. By varying the coating conditions, it was possible to produce a uniform polydopamine coating with tunable thickness, which acted as an additional barrier to reduce burst release and achieve a more sustained release profile.

## 1 Introduction

Considerable research efforts have been made to produce porous materials for drug delivery.^1^ Due to high surface area, large porosity, and facile surface functionalization, porous materials have been widely employed to carry a variety of drugs, proteins, and genes.^2^ Among various porous materials, nanofibers are highly attractive in the field of drug delivery system.^3^ In addition to the high surface-to-volume ratio and possible intrinsic pores within the nanofibers, the highly interconnected porosity generated by stacking or network of nanofibers is considerably beneficial for tissue engineering and controlled drug release.^4^ The common method to prepare nanofibers is electrospinning,^5^ which has been frequently used to develop polymer and nanocomposite nanofibers for biomaterials and biomedical applications.^6−8^ In spite of many benefits and advantages of the electrospinning method, there are still some challenges to be addressed, such as the difficulty in increasing the production scale and the heavy use of volatile and toxic organic solvents.^9,10^

Ice templating is a versatile approach to fabricating nanofibers for the applications of drug delivery, tissue engineering, and implants.^11,12^ Ice templating can be readily applied to aqueous solutions or suspensions where ice is formed under freezing conditions, excluding solutions or particles from the growing ice crystals. The subsequent removal of ice crystals, usually by freeze-drying, generates highly interconnected porous structures.^12,13^ With the advantages of tunable microstructure, diverse employment of materials, and easy scalability, ice templating has drawn extensive attention and can be an effective method for large-scale nanofiber preparations.^12,14,15^ More importantly, when a very dilute polymer solution is employed, polymeric nanofibers can be formed, with the fiber diameter impacted mainly by the polymer concentration or freezing condition.^12^

A number of synthetic and natural polymers have been utilized to produce fibrous materials via ice templating for pharmaceutical applications. Among these polymer materials, poly(vinyl alcohol) (PVA) is attractive and has gained huge interests.^16^ Because of its desirable properties such as biocompatibility, nontoxicity, and hydrophilicity, PVA has been widely employed for cartilage tissue engineering,^17^ wound dressing,^18^ and drug delivery.^19^ PVA nanofibers prepared by ice templating play a significant role in the field of controlled drug release. Given their desirable properties such as high surface area-to-volume ratio, bioadhesiveness, hydrophilicity, and chemical resistance, PVA nanofibers have been employed for encapsulating and protecting proteins from harsh or unfavorable environments. PVA nanofibers made by freeze-drying are susceptible to moisture and can be readily dissolved in water. This is a disadvantage when utilizing PVA nanofibers as drug carriers or scaffolds, since PVA nanofibers are dissolved immediately in aqueous surroundings, resulting in a burst drug release. It is thus necessary to carry out modifications of PVA nanofibers to improve the stability in a wet environment. Cross-linking PVA is a common modification approach to render stable PVA nanofibers, enhancing the biochemical properties.^20^ However, there are limitations with the common PVA cross-linker, glutaraldehyde. Although the glutaraldehyde cross-linking method is highly efficient, the undesirable properties such as high vapor pressure, pungent odor, and low biocompatibility/carcinogenicity limited its applications in biomedical and pharmaceutical fields.^21^

Polydopamine (PDA) coating is regarded as a material-independent toolbox for surface modification and functionalization, inspired by the unique adhesion ability of marine mussels on virtually any substrate. Dopamine (DA) possesses similar functional groups and adhesive mechanism to 3,4-dihydroxy-l-phenylalanine (DOPA), which is proved to be the main protein component of mussels and plays a key role during the adhesion process.^22^ PDA coating involves the oxidation polymerization of DA and surface adhesion. Although the debate about the mechanism of DA polymerization remains, all the polymerization mechanisms seem to involve autoxidation, intermolecular rearrangement of DA monomers, and polymerization of these DA monomers.^23^ With the unique adhesion ability, excellent biocompatibility, and great biodegradability, polydopamine surface functionalization exhibits great potentials for biomedical and pharmaceutical fields.^24^ Polydopamine has been widely employed to form a coating on diverse materials for biomedical uses, for instance, working as a photothermal therapy agent on silica nanoparticles for cancer therpy,^25^ functionalizing polymeric nanoparticles with ligands to develop a targeted deliver system for the treatment of liver cancer,^26^ and modifying sodium alginate-based scaffolds for tissue engineering applications.^27^

Capable of moderating biological activities and cellular pathways in which small molecular drugs may not be able to, protein therapeutics have been intensively investigated.^28^ One of the biggest challenges in this area is achieving a high loading efficiency while not destabilizing the unique structures of proteins.^29^ To address this issue, considerable efforts have been made to encapsulate protein via different techniques. For instance, bovine serum albumin (BSA) was encapsulated into poly(dl-lactic-*co*-glycolic acid) (PLGA) nanoparticles by double emulsion solvent evaporation.^30^ Dextran sulfate/poly l-arginine-based microcapsules were employed to encapsulate human serum albumin via CaCO_3_ particle-templating and layer-by-layer assembly.^31^ Lysozyme was loaded on poly(ε-caprolactone) (PCL) and poly(ethylene oxide) (PEO) nanofibrous meshes through electrospinning methods.^32^ However, most of these methods involve the use of organic toxic solvents (e.g., dichloromethane, chloroform, and dimethyl sulfoxide) in the encapsulation process, thereby triggering protein denaturation to a certain extent. Furthermore, some of the protein delivery systems with no use of organic solvents tend to give low encapsulation efficiency and low loading, such as liposomes and polymersomes with vesicular structures.^29,33,34^ Hence, it is essential to develop a novel protein delivery system where toxic organic solvents are not used in the encapsulation process, while a high loading efficiency is achieved.

Herein, we report the development of PVA/BSA nanofibers via ice templating, achieving a high loading of protein in hydrophilic nanofibers. The resulting composite nanofibers are further modified by PDA coating for the controlled release of BSA. Compared to the most existing protein delivery systems, our study utilizes only water in the encapsulation process, while a 100% loading efficiency is achieved without any waste of protein drugs. Due to the issue of the dissolution of PVA nanofibers in aqueous solution, PDA coating is performed on PVA nanofibers in a system with organic solvent and organic base, which also prevents the leaking of BSA during the coating process. To the best of our knowledge, this is a novel approach to encapsulating a protein in polymer nanofibers via ice templating and coating polymer nanofibers in an organic system without the use of an aqueous buffer solution. A uniform PDA coating forms on PVA nanofibers and PVA@BSA nanocomposites, which not only improves the stability of hydrophilic PVA nanofibers in aqueous medium but also serves as a barrier for controlling the BSA release and reducing the burst release with good biocompatibility.

## 2 Materials and Methods

### 2.1 Chemicals and Materials

Poly(vinyl alcohol) (89–98 kDa, 99+% hydrolyzed), dopamine hydrochloride, piperidine, bovine serum albumin (≥96%), phosphate-buffered saline tablets (pH 7.2–7.6, 1 tablet/200 mL), cell proliferation kit I (MTT or 3-(4,5-dimethylthiazol-2-yl)-2,5-diphenyltetrazolium bromide), and Bradford reagent (for 0.1–1.4 mg/mL protein) were purchased from Sigma-Aldrich. Glutaraldehyde (25% aqueous solution) and hydrochloric acid (37%) were provided by Alfa Aesar. Ethanol, Dulbecco’s modified Eagle's medium (DMEM), and fetal bovine serum (FBS) were supplied by Fisher Scientific. All chemicals were of analytical grade and used without further treatment.

### 2.2 Preparation of PVA Fibers and PVA@BSA Composites

0.1 g PVA was dissolved in 100 mL of DI water by continuously stirring at 80 °C to form 0.1 wt % PVA solution.^35^ After cooling to room temperature, the PVA solution was frozen in liquid nitrogen and then freeze-dried for 48 h in a freeze-dryer (CoolSafe, Jencons-VWR) to produce PVA fibers. In order to prepare PVA@BSA composite fibers, an aqueous BSA solution was first prepared by dissolving 0.2 g of BSA in 100 mL of DI water at room temperature. The PVA solution and BSA solution were then mixed at mass ratios (PVA: BSA) of 1:1, 2:1, and 1:3 at room temperature. Finally, PVA/BSA solutions were frozen in liquid nitrogen and freeze-dried for 48 h.

### 2.3 PDA Coating and Glutaraldehyde Cross-linking of PVA Fibers and PVA@BSA Composites

Dopamine hydrochloride was dissolved in 24 mL of ethanol to form solutions with different concentrations (0.77, 1.54, and 3.85 mg/mL). Piperidine was then added at a molar ratio of 2:1 to dopamine hydrochloride.^36^ Afterward, PVA fibers or PVA@BSA composite fibers were placed in the DA/piperidine solution for 4 h at room temperature and then transferred to a freezer (−20 °C) for 17 h. In order to investigate the effects of coating conditions, PVA fibers were also placed in 0.77 mg/mL DA/piperidine solution at room temperature for 21 h, in freezer (−20 °C) for 21 h, and their combination (room temperature for 4 h and then in a freezer for 17 h). PVA fibers were also coated in 0.77 mg/mL DA/piperidine solution at different volumes (3, 7, and 24 mL) for 4 h at room temperature and then transferred to the freezer (−20 °C) for 17 h. The fibrous materials were then taken out and washed by ethanol three times. They were finally placed in a vacuum oven at room temperature for 24 h.

As a comparison, PVA@BSA composites (1:1) were also treated for 20 h by glutaraldehyde vapor cross-linking at room temperature in a seal glass vessel, where 12 mL of glutaraldehyde (25% aqueous solution) was mixed with 1 mL of hydrochloric acid (37%).

### 2.4 Cell Culture and Cytotoxicity Assay

Lung cells (A549) were maintained in DMEM supplemented with 10% fetal bovine serum. Cells were maintained in a 5% CO_2_ incubator at 37 °C.

Cell viability was evaluated by using MTT assay. A549 cells were seeded at a concentration of 5 × 10^4^ cells/well in a 100 μL culture medium and incubated (5% CO_2_, 37 °C) for 24 h. For the PDA nanoparticles, the culture medium was removed and replaced with 90 μL of culture medium and 10 μL of nanoparticles dispersed in water (final concentration of nanoparticles: 1–512 μg/mL). For the PDA@PVA@BSA (2:1) nanofiber, the culture medium was removed and replaced with 100 μL of fresh culture medium and approximately 1 mg of nanofiber. The cells in the presence of the samples were incubated for 24 h (5% CO_2_, 37 °C). After 24 h of incubation, the media within each well was removed and replaced with 100 μL of culture medium and 10 μL of the MTT labeling reagent (final concentration of 0.5 mg/mL). The microplate was incubated for 4 h (5% CO_2_, 37 °C). One hundred microliters of the solubilizing buffer was added to each well, and the microplate was allowed to stand overnight in the incubator. The solubilization of the purple formazan crystals was measured at an absorbance wavelength of 570 nm. All samples were tested in triplicate.

Statistical analysis was conducted using one-way analysis of variance (ANOVA), followed by Tukey's post hoc test. Differences were deemed as statistically significant if a value of *p* < 0.05 was obtained.

### 2.5 Drug Release Monitored by Bradford Assay

Around 18 mg PDA@PVA@BSA composites was placed in 15 mL of PBS at room temperature. 0.05 mL of PBS was withdrawn at different time intervals and then mixed with 1.5 mL of Bradford reagent. After 6 min, 0.1 mL of solution from the Bradford reagent with PBS was placed in a 96-well plate for UV–vis test at the wavelength of 596 nm.

### 2.6 Characterization

The morphology of PVA fibers and PDA@BSA composites was analyzed by a scanning electron microscope (Hitachi S4800 SEM). Samples for SEM observations were prepared by sticking them on carbon tabs and coated with gold with a 15 mA sputter current for 45 s. A Vertex 70 fourier transform infrared (FTIR) spectrometer was used to analyze the functional groups. The amount of PDA coating on PVA@BSA composites was determined by means of a thermogravimetric analysis system (Netzsch TG 209 F1 Libra, TGA), CHN analysis, and energy-dispersive X-ray (EDX) analysis. TGA was performed with a heating rate of 25–900 °C/min in nitrogen. An MTT cytotoxicity assay was used for estimating the cytotoxic properties of these materials by using a microplate (ELISA) reader (ThermoFischer Scientific Varioskan Lux). The release of BSA after treatment with the Bradford reagent was determined by using a UV–vis plate reader (Bio-Tek uQuant).

## 3 Results and Discussion

### 3.1 PVA Fibers to PDA@PVA Fibers

**Figure 1:**
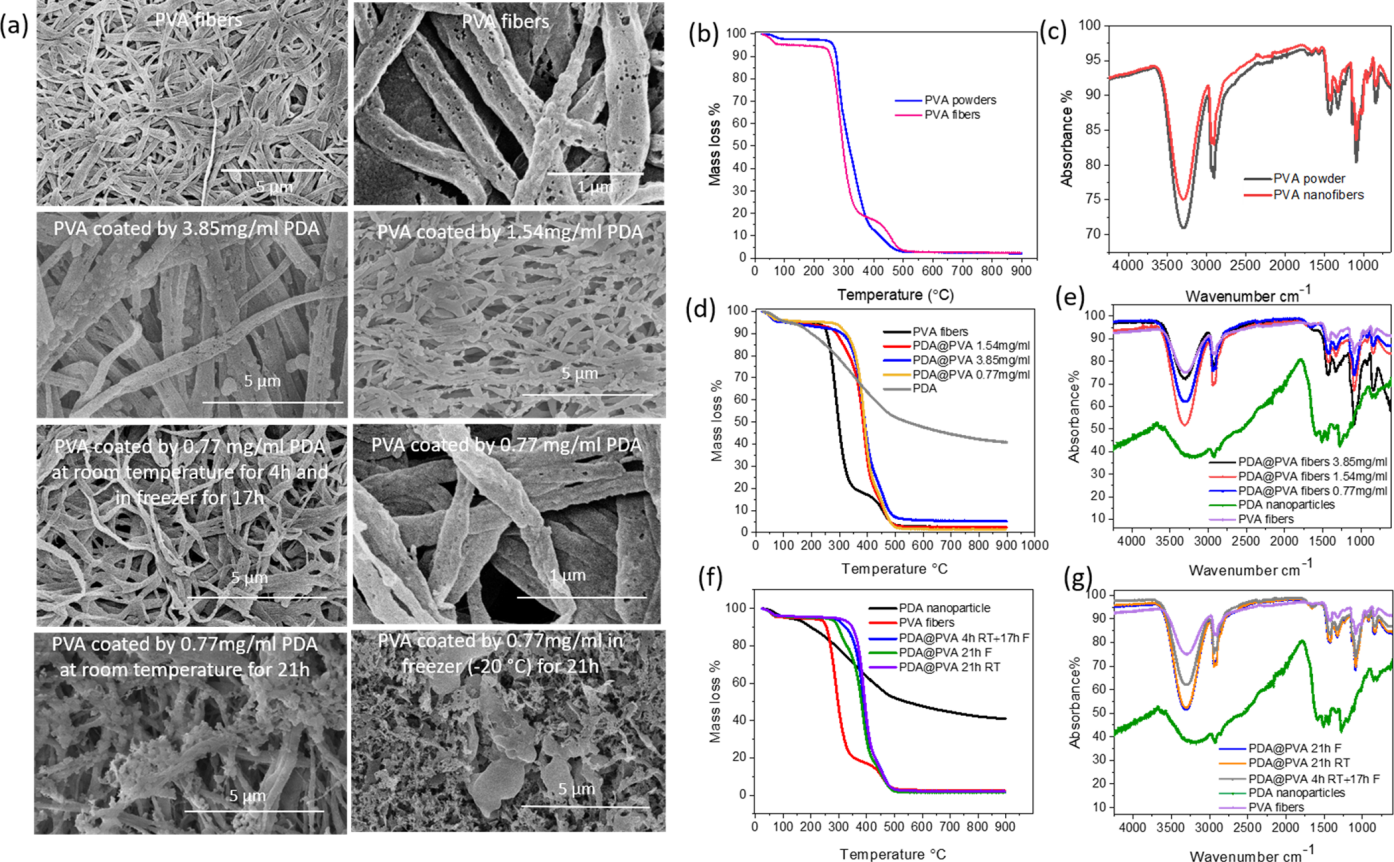
(a) Morphology of PVA nanofibers and PDA-coated PVA nanofibers at different coating conditions. (b, c) TGA profiles and FTIR spectra of PVA powder and PVA fibers. (d, f) TGA profiles and (e, g) FTIR spectra of PVA fibers coated by PDA at different DA concentrations and coating temperatures.

PVA nanofibers were produced by ice templating from an aqueous PVA solution (0.1 wt %). The nanofibers were observed by SEM, as shown in Figure 1a. From TGA, the decomposition temperatures of both PVA powders and nanofibers were found to be around 260 °C, and the mass loss kept constant at 900 °C with the residual mass at around 2.35% (Figure 1b).^37,38^ When the FTIR spectra of PVA powders and PVA nanofibers were compared, no new peaks were observed (Figure 1c). This is expected because there is no chemical reaction involved in the ice-templating process. Polydopamine was synthesized in a system consisting of an organic solution and an organic base. Specifically, dopamine was dissolved in ethanol forming a 5 mg/mL solution. Piperidine (2:1 molar ratio to DA) was then added into that solution to deprotonate or oxidize dopamine, subsequently producing polydopamine.^36^ The resulting material showed the formation of aggregated PDA particles (Figure S1a,b). The TGA curve of PDA was totally different from that of DA (Figure S1c). The decomposition temperature of DA was about 300 °C, and the residual mass was around 16% at 900 °C. As for PDA, water and the residual solvent were eliminated at 0–150 °C.^39^ PDA exhibited a significant mass loss between 150 and 500 °C, which could be attributed to the degradation of the phenolic hydroxyl group and the decomposition of the ring structure.^40−43^ The residual mass of PDA was 40% at 900 °C, which was much higher than that of DA (Figure S1c). From the FTIR spectra of PDA, it is observed that the band at 3338 cm^–1^ was attributed to the N–H stretching vibration.^44^ The bands at 3153 and 3043 cm^–1^ resulted from the aromatic O–H stretching vibration (Figure S1d).^45^ The bands of O–H were located at 2929 and 2864 cm^–1^, which shifted into a lower wavenumber compared to the bands of O–H for dopamine. The new bands at 1514 and 1450 cm^–1^ were assigned to the stretching vibrations of indole and indoline structures of PDA.^46−48^

Three different concentrations (3.85, 1.54, and 0.77 mg/mL) of DA solutions were employed to coat PDA on PVA nanofibers at room temperature for 4 h and then in a freezer at −20 °C for 17 h. The PDA-coated PVA fibers started to decompose at approximately 330 °C, which was higher than the decomposition temperature of untreated PVA fibers (260 °C) (Figure 1d). The residual mass of PVA coated by 3.85 mg/mL PDA was 5.11% at 900 °C, which was higher than that of untreated PVA fibers (2.35%). For the PVA fibers coated by 0.54 and 0.77 mg/mL, the residual masses were 2.38 and 2.37%, respectively, which were close to that of untreated fibers. Peaks of untreated PVA fibers at 2943, 2904, 1429, and 1340 cm^–1^, which were attributed to the symmetric stretching of CH_2_ and OH bending,^49,50^ were mostly overlapped with the peaks from PDA. Thus, FTIR curves of PVA fibers coated by PDA were similar to that of untreated PVA fibers (Figure 1e). Furthermore, the microstructure of PVA fibers coated by PDA was assessed by SEM. For the PDA@PVA fibers (3.85 mg/mL), more PDA nanoparticles gathered on PVA fibers or clumped together, whereas less PDA nanoparticles were observed from PDA@PVA fibers (1.54 mg/mL) (Figure 1a). When the PVA fibers were coated by 0.77 mg/mL PDA, a smooth fibrous structure could be observed, as in Figure 1a.

The effect of the coating temperature was then investigated. PVA fibers were coated at different temperatures (room temperature, freezer −20 °C, and their combination) with the same DA concentration (0.77 mg/mL) in 24 mL of ethanol and 2:1 molar ratio of piperidine and DA. When the coating reaction was conducted at room temperature for 21 h, the resulting PDA@PVA fibers began to decompose at 330 °C (Figure 1f). When the PVA fibers were coated at room temperature for 4 h and then in the freezer for 17 h, the decomposition temperature was 300 °C. The decomposition temperature of the PDA-coated PVA fibers prepared only in the freezer for 21 h was reduced further to 280 °C. This confirms that DA polymerization has occurred. However, at a lower reaction temperature, the degree of polymerization/cross-linking is likely to be lower, resulting in a lower decomposition temperature, as characterized by TGA. This is also consistent with the FTIR analysis, i.e., similar FTIR profiles of PVA fibers coated by PDA at different temperatures were observed (Figure 1g). However, the microstructures were different for the PVA fibers coated at different temperatures. Small PDA nanoparticles clustered together, forming an uneven PDA coating layer for the PDA-coated PVA nanofibers prepared at room temperature (Figure 1a). When coating PDA on PVA fibers in a freezer for 21 h, PDA nanoparticles were more prone to bind together to form large agglomerates between PVA fibers. A uniform coating of PDA on PVA fibers was achieved when the coating reaction was performed 4 h at room temperature and then 17 h in a freezer (Figure 1a).

**Figure 2:**
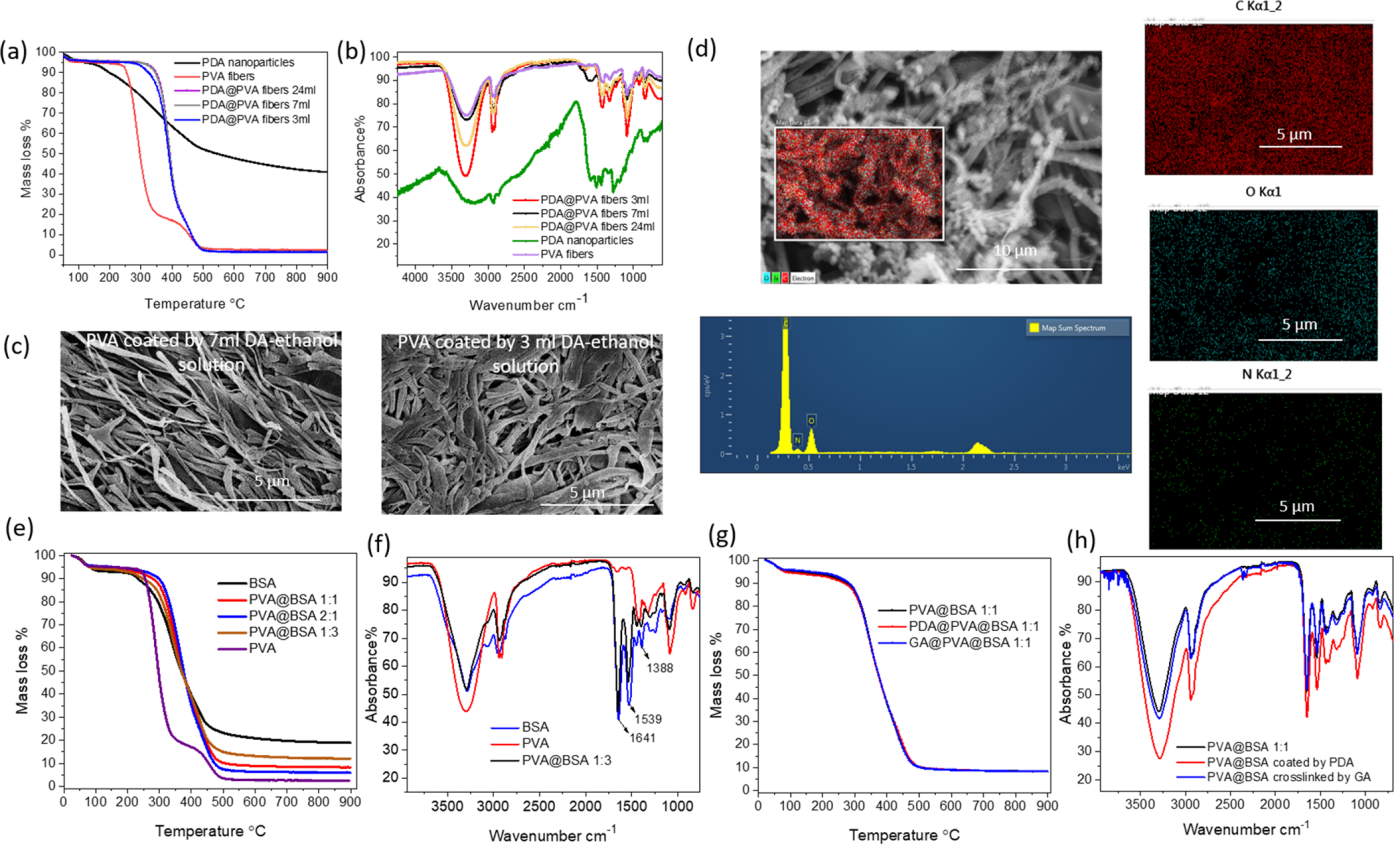
(a) TGA profiles and (b) FTIR spectra of PVA fibers coated by PDA with different volumes of DA-ethanol solutions. (c) Morphology of PDA-coated PVA nanofibers at different coating volumes. (d) EDS analysis of PDA-coated PVA nanofibers prepared from 3.85 mg/mL DA-ethanol solution. (e) TGA profiles and (f) FTIR spectra of PVA, BSA, and PVA@BSA with different mass ratios. Comparison of TGA profiles (g) and FTIR spectra (h) of PVA@BSA (1:1) and after coating with PDA or cross-linking by GA.

We further investigated the effect of the volume of the DA-ethanol solution, which changed the mass ratio between DA and PVA fibers. The volume of the DA-ethanol solution (concentration 0.77 mg/mL) was varied to 3, 7, and 24 mL, respectively, while keeping other coating conditions unchanged (4 h at room temperature and 17 h in a freezer). TGA showed that the change of the DA-ethanol solution volume did not change the decomposition temperature significantly while the degree of mass loss seemed very similar (Figure 2a). This suggests that the mass ratio of DA to PVA fibers was not a limiting factor under the studied reaction conditions. All the PDA-coated PVA nanofibers prepared in different amounts of DA-ethanol solution showed similar FTIR spectra and smooth fibrous structure (Figure 2b,c).

The PDA coating of the PVA fibers was also confirmed by elemental analysis. Untreated PVA fibers gave a carbon content of 50.68% with no nitrogen, while the analysis of PDA nanoparticles showed a C content of 58.98% and a N content of 9.63% (Table S1). When the PVA fibers were coated in the DA solution with a concentration of 3.85 mg/mL, the carbon content was slightly increased to 52.64%. Together with the confirmed N content of 0.33%, it indicated the successful coating of PDA onto the PVA nanofibers. The EDX analysis of the PDA-coated PVA fibers exhibited uniform distribution of detected elements across the nanofibers (Figure 2d). Small green dots show mapping for nitrogen. The nitrogen mass content and atom content were 0.61 and 0.64%, respectively (Table S2). These values are higher than that of the microanalysis. This is because EDX is a surface analysis with a certain penetration depth, while microanalysis is the result of the whole material. Since PDA was coated on PVA nanofibers, it is expected to have a higher PDA content (and hence N content) on the surface. It was also noted that the PDA-coated fibers prepared from the DA-ethanol solutions with lower concentrations showed a smaller increase in C content, but no N was detected. This likely resulted from a thin coating of PDA, nonuniform distribution of PDA nanoparticles, or very low mass ratio of PDA to PVA across the materials. The coating temperature and concentration of DA influenced the size of PDA nanoparticles, thus resulting in different coating effects.^51^ The nucleation rate of PDA was higher at room temperature than that in the freezer, likely leading to smaller nanoparticles gathering on PVA fibers at room temperature and larger PDA lumps between PVA fibers in the freezer (Figure 1a).^52,53^ Larger sized PDA nanoparticles on PVA fibers formed when coating in 3.85 mg/mL than that in 1.54 mg/mL, as in Figure 1a. The generation of quinone played an important role in the PDA synthesis. Quinone formed quickly at a higher DA concentration and was trapped by DA at the same time, which resulted in slower nucleation formation rate and particle growth rate.^54^

#### 3.1.1 In Situ Loading of BSA on PVA and Subsequent PDA Coating

BSA was loaded directly on PVA during the ice-templating process. Aqueous solutions containing BSA and PVA were frozen in liquid nitrogen and then freeze-dried to produce PVA fibers with BSA. Since there was no loss of BSA during this process, a 100% loading efficiency of BSA was achieved. The successful loading of BSA on PVA was confirmed by TGA, FTIR, CHN, and SEM analyses. TGA showed that the residual mass of BSA was around 18.86%, when the temperature reached 900 °C, which was much higher than 2.35% of untreated PVA fibers (Figure 2e). The residual mass of PVA@BSA (1:3) at 900 °C was the highest (12.01%), followed by PVA@BSA (1:1) and PVA@BSA (2:1), with 8.23 and 5.98%, respectively (Figure 2e). Furthermore, new peaks related to BSA were observed from the FTIR spectra of PVA@BSA composite fibers. The peaks at 1388, 1398, and 1413 cm^–1^ were attributed to the vibration of amide III.^55^ The bands at 1539, 1512, and 1531 cm^–1^ were related to amide II.^56^ Additionally, the new peaks were also present at 1641, 1635, and 1651 cm^–1^, which were attributed to amide I (Figures 2f and S2a).^57^ These peaks indicated the successful loading of BSA on PVA.

The PVA@BSA fibers prepared by freeze-drying can dissolve instantly in aqueous medium. It is therefore unsuitable to be used as a scaffold for the release of BSA. Based on the results obtained above from coating PVA fibers with PDA, the same coating process (i.e., 0.77 mg/mL of DA-ethanol solution at room temperature for 4 h and then in a freezer for 17 h) was applied to coat the PVA@BSA fibers. This would improve the scaffold stability and also allow the PDA coating acting as a barrier to control the release of BSA. Further characterization by TGA showed that the PDA-coated PVA@BSA fibers exhibited similar TGA profiles with close residual mass before and after PDA coating of PVA@BSA (1:1, 2:1, and 1:3) (Figures 2g and S2b,c), indicating a thin PDA coating on the PVA@BSA fibers. Furthermore, the peaks at amide I, amide II, and amide III remained after PDA coating (Figures 2h and S2d). This suggested that the PDA coating treatment did not destroy the structure of BSA. The long-term stability of PVA@BSA coated with PDA was also investigated. After being soaked in water for 7 months, the PDA@PVA@BSA materials (1:1, 2:1, and 1:3) were still intact without breaking into fragments or dissolving (Figure S3a). The FTIR results showed that the peak at around 1540 cm^–1^ (attributed to amide II vibrations) disappeared, and the band at 1330 cm^–1^ (belonging to OH stretching) was enhanced for PDA@PVA@BSA (1:1, 2:1, and 1:3) (Figure S3b–d), indicating that BSA was released while the matrix fiber structure was stable in water for a long time.

An MTT assay was performed to investigate the cytotoxicity of the PDA coating on PVA@BSA. A549 cells were treated with PDA nanoparticle concentrations of 1–512 μg/mL (Figure S4a). More than 73% cell viability was observed at all nanoparticle concentrations tested. Cells treated with the highest nanoparticle dose of 512 μg/mL were found to have a cell viability of 86 ± 9% (*p* > 0.05 compared to untreated control). No clear dose-dependent toxicity was observed at the nanoparticle concentrations tested, consistent with other studies.^58^ Treatment of A549 cells with PDA@PVA@BSA (2:1) gave a cell viability of 76 ± 15% (*p* = 0.002) (Figure S4b). Literature studies show that both PVA as a biocompatible polymer and BSA have no cytotoxic effect on healthy cells.^59^ Due to the insolubility and nonwater dispersibility of PDA@PVA@BSA (2:1) nanofibers, it is possible that the cell viability results were impacted by the sedimentation of the fibers that may have affected oxygenation of the cells.

An alternative approach to cross-linking PVA using glutaraldehyde was investigated. Usually, this type of cross-linking is carried out in aqueous glutaraldehyde (GA), with acid as a catalyst. However, because PVA@BSA is soluble in water, a vapor cross-linking process was used. A glutaraldehyde solution with a small amount of HCl was added into a glass bottle. A scaffold or support was placed above the glutaraldehyde solution, with the PVA@BSA fibers placed on the scaffold. The glass bottle was then sealed to allow the vapor cross-linking reaction to occur at room temperature. The resulting material became a bit more rigid and became insoluble and nonshrinking in water. The TGA profile and the FTIR spectrum were very similar to those of PVA@BSA or PDA-coated PVA@BSA fibers (Figure 2g,h).

**Figure 3:**
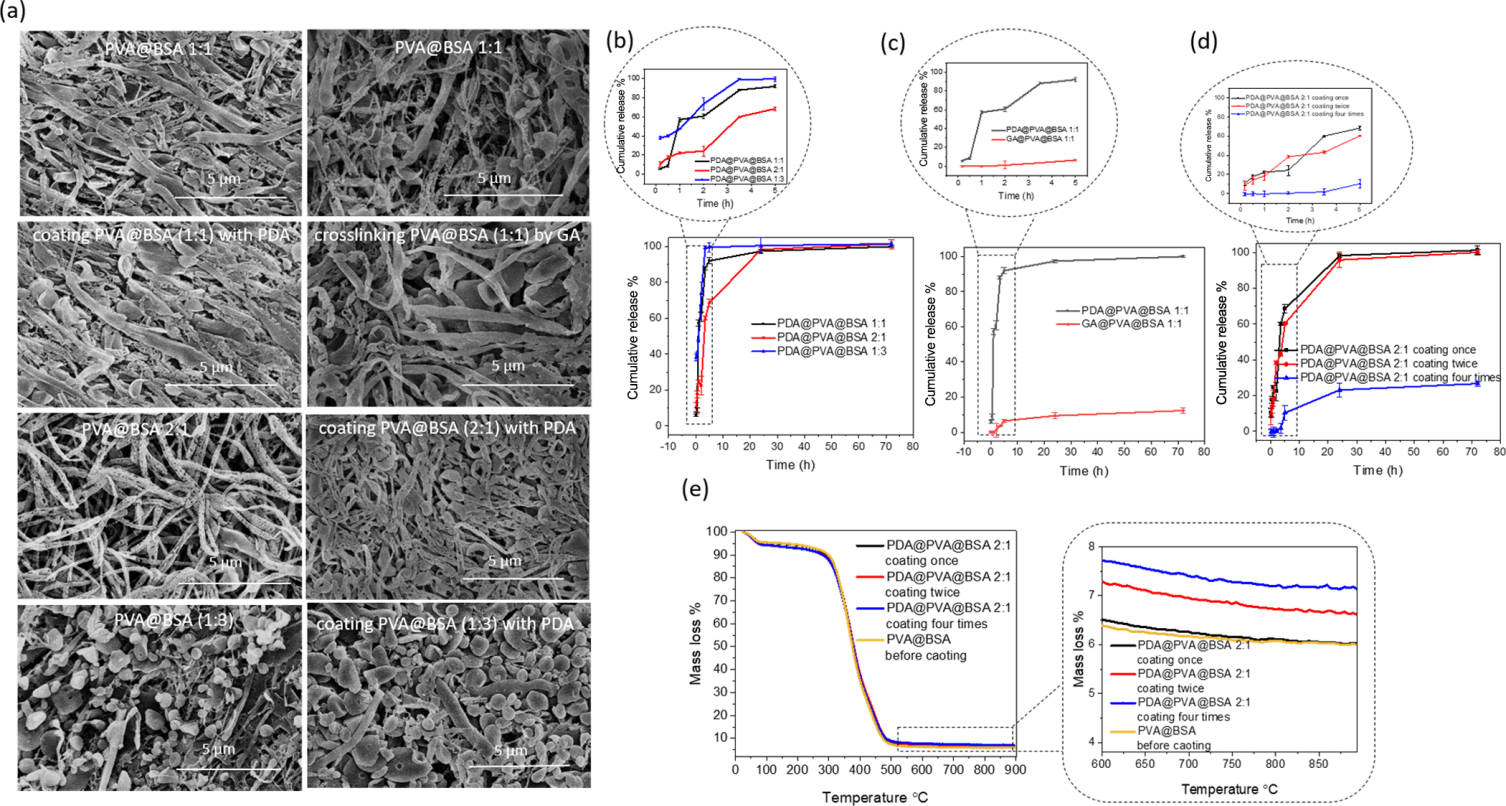
(a) Morphology of PVA@BSA after the coating with PDA or cross-linking with GA. The cumulative release of BSA from PDA@PVA@BSA with different mass ratios (b) and the comparison with PVA@BSA (1:1) treated by GA (c). (d) Cumulative release of BSA from PDA@PVA@BSA with different coating times and (e) their TGA profiles.

The coating of PVA@BSA (1:1) by PDA increased the N content from 6.29 to 7.01% (Table S3). Similarly, the nitrogen contents of PDA@PVA@BSA (2:1) and (1:3) were 4.53 and 10.73%, which were larger than 4.18 and 9.87% of PVA@BSA (2:1) and (1:3), respectively. The microstructure of the resulting materials was further examined by SEM. When the mass ratio between PVA and BSA was 1:1, nanofibers and nanoparticles were both observed (Figure 3a). Both PDA coating and GA vapor cross-linking did not change or damage the morphology (Figure 3a). With the increase of the mass ratio of PVA, PVA@BSA (2:1) presented a nanofibrous structure, and this fibrous structure remained after PDA coating. However, more nanoparticles were observed for PVA@BSA (1:3) (Figure 3a). After coating with PDA, these nanoparticles were still present (Figure 3a). These results indicated the successful BSA loading on PVA and PDA coating/GA cross-linking of PVA@BSA composites. Further, the morphology of PVA@BSA was not changed by PDA coating or GA cross-linking.

### 3.2 BSA Release Behavior

The PDA-coated or GA-cross-linked PVA@BSA materials were stable in aqueous medium. The high loading capacity and loading efficiency of BSA (as a model protein) in hydrophilic fibers could provide a potentially powerful platform for protein release. As such, the BSA release behavior was investigated. Among PDA@PVA@BSA with mass ratios of 1:1, 2:1, and 1:3, BSA release from PDA@PVA@BSA (1:3) was the fastest, followed by PDA@PVA@BSA (1:1) and PDA@PVA@BSA (2:1), respectively (Figure 3b). The time to reach a plateau release varied from about 3.5 to about 25 h. The release rate looks related to the loading level of BSA in the materials. A higher loading of BSA resulted in a faster release (Figure 3b). As mentioned earlier, two methods (PDA coating and GA cross-linking) were used to make PVA@BSA stable in aqueous medium. Figure 3c shows a very different release profile between the two resulting materials. Notably, the BSA release from GA-cross-linked PVA@BSA (1:1) was very slow, only reaching a cumulative percentage release of 9.41% after 70 h. This may be potentially useful for a long-term release. In comparison, PDA@PVA@BSA (1:1) displayed a gradual release profile within 5 h and a slow release process until it reached 100% release at 72 h. The slow release of BSA from the GA-cross-linked material is highly likely due to the cross-linking of BSA by GA. In this regard, it is possible to tune the BSA release by varying the GA cross-linking condition and/or the degree of BSA cross-linking.

The influence of the coating times on the BSA release behavior was then investigated. PVA@BSA (2:1) was coated once, twice, and four times under the same coating conditions, respectively. As shown in Figure 3d, the slowest release of BSA is from PVA@BSA coated by PDA four times, and the cumulative release percentage was around 26.69% at 72 h. Compared with PVA@BSA coated by PDA one time, BSA from PVA@BSA coated by PDA twice showed a slower release behavior but reached 100% cumulative release at 72 h. Compared to the release percentage, the difference in the released amount of BSA from the PVA@BSA samples coated with PDA was more obvious (Figure S5a). The release from PVA@BSA coated by PDA four times was the lowest in comparison to PVA@BSA coated by two times and one time. The elemental and thermalgravimetric analyses were further performed to characterize PVA@BSA (2:1) coated by PDA for different coating times. With the increase of coating times, the residual mass became larger at 900 °C, with 7.13, 6.60, and 6.01% (Figure 3e). Similarly, the nitrogen content was also higher as the coating times increased. The highest nitrogen content of PVA@BSA coated by PDA four times was 4.93%, followed by 4.68% of PVA@BSA coated by PDA twice and 4.53% of PVA@BSA coated by PDA once (Table S4). Thus, the thickness of the PDA coating layer could be adjusted on PVA@BSA composites with changing coating times, resulting in a slower BSA release behavior.

**Figure 4:**
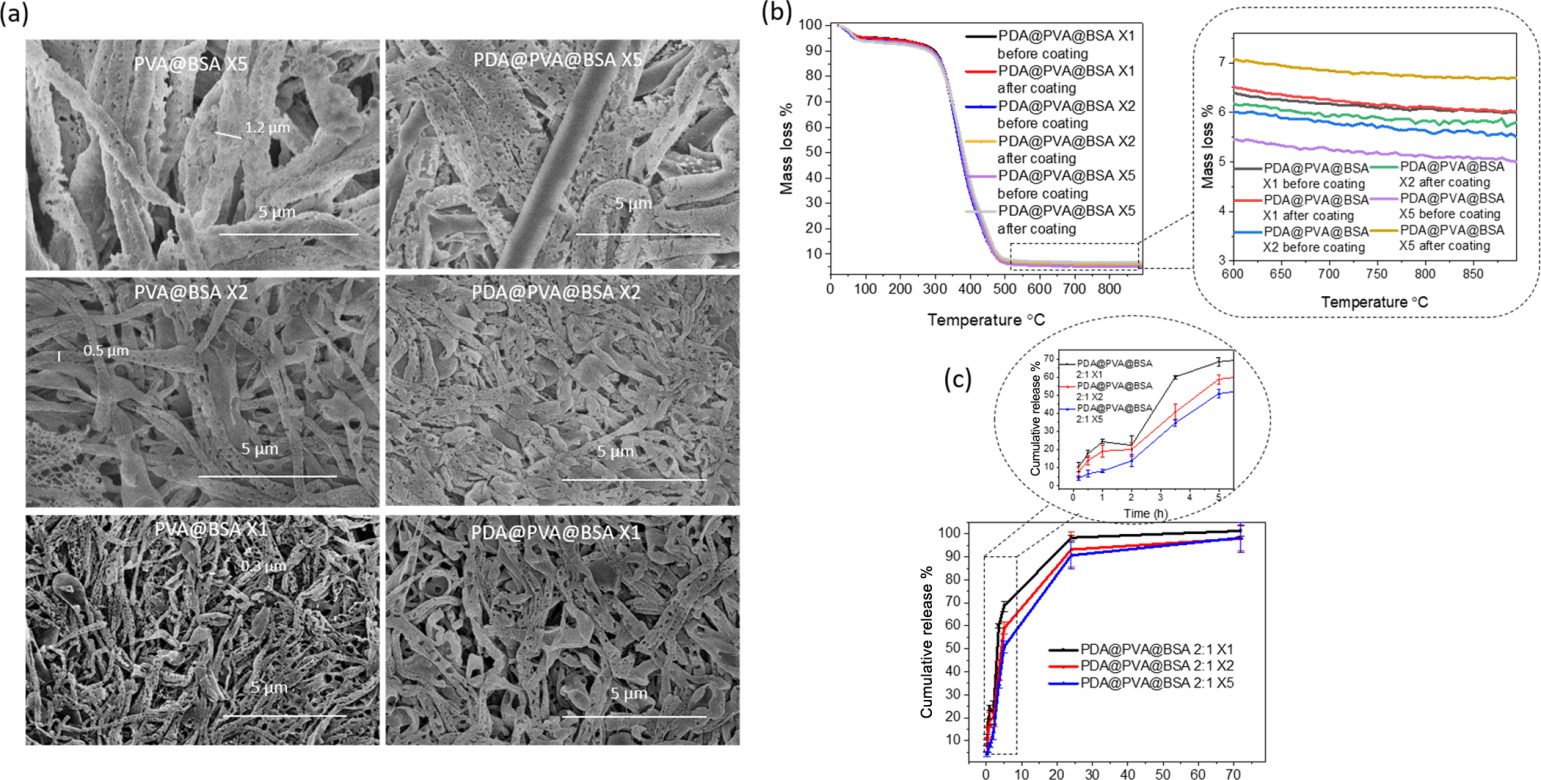
(a) Morphologies of PVA@BSA prepared from aqueous solutions of PVA and BSA with different concentrations with the mass ratio of PVA:BSA = 2:1 and their subsequent coating by PDA. (b) TGA profiles and (c) cumulative release of BSA from PDA@PVA@BSA prepared with different concentrations of PVA and BSA solutions.

The PVA@BSA materials were also prepared by using different concentrations of PVA and BSA while keeping the mass ratio of PVA:BSA = 2:1. Their microstructures and PDA coating thickness were determined by SEM and TGA at first. The PVA@BSA fibers became longer and thicker with increasing concentration. When 20 mL of 5 mg/mL PVA solution was mixed with 5 mL of 10 mg/mL BSA solution (named PVA@BSA X5), the composite consisted of long fibers with the diameter of around 1.2 μm (Figure 4a). After being coated by PDA in standard conditions (0.77 mg/mL DA/ethanol/piperidine at room temperature for 4 h and then in a freezer for 17 h), they remained fibrous, and the diameter was similar to those before the coating treatment (Figure 4a) (named PDA@PVA@BSA X5). With the concentration decreasing to 2 mg/mL of PVA solution and 4 mg/mL of BSA solution (named PVA@BSA X2), the diameter of PVA@BSA composite fibers reduced to around 0.5 μm, as shown in Figure 4a, which was thinner than that of higher concentration.

Similarly, PDA coating did not damage the fibrous structure and increase the diameter of fibers either for PDA@PVA@BSA X2 (Figure 4a). The PVA@BSA fibers were much thinner when the concentration was 1 mg/mL of PVA and 2 mg/mL of BSA solution (named PVA@BSA X1), with the diameter of approximately 0.3 μm (Figure 4a). After the PDA coating treatment, fibers (PDA@PVA@BSA X1) looked similar to PVA@BSA X1 as well. As shown in Figure 4b, the residual mass of PVA@BSA X5 before and after PDA coating was 5.01 and 6.71% at 900 °C, respectively. As for PVA@BSA X2, the residual masses without and with PDA coating were 5.47 and 5.82%, followed by 5.99 and 6.03%. Thus, the thickest and thinnest PDA coating layers were developed on PVA@BSA X5 and PVA@BSA X1, respectively, which was subsequently confirmed by the BSA release test. PDA@PVA@BSA X5 presented the slowest BSA cumulative release and concentration within the first 5 h, and 97.96% of BSA was released at 72 h (Figures 4c and S5b). Additionally, PDA@PVA@BSA X1 showed the fastest BSA release profile, with 100% of BSA released at 72 h.

## 4 Conclusions

An ice-templating method was used to prepare hydrophilic polymer nanofibers with the in situ uploading of proteins. As demonstrated in this study, PVA nanofibers with incorporated BSA at a 100% loading efficiency were produced. In order to improve the fiber stability and allow for better control for the release of BSA, a thin and uniform PDA coating was formed on the freeze-dried nanofibers in an organic coating system, which was composed of ethanol and piperidine. Coating temperature and DA concentration played important roles in adjusting the size and distribution of PDA nanoparticles on PVA fibers. The controlled release of BSA from the PDA-coated PVA@BSA nanofibers was demonstrated, with the release profiles being controlled by tuning the PDA coating thickness and concentration of PVA and BSA during the preparation process. It should be noted that the PDA coating method offered less use of toxic chemicals and a relatively easier controlled BSA release profile than the alternative glutaraldehyde cross-linking method. We believe that this approach can be readily extended to other polymer/protein systems and subsequently achieve desirable protein loading and controlled release behavior, depending on the protein size, charge, and interaction with the polymer fiber matrix.

## Supporting Information Available

SEM analysis of PDA nanoparticles; TGA profiles and IR spectra of DA and PDA; CHN elemental analysis results of PVA fibers, PDA nanoparticles, and PVA coated by PDA in different conditions; EDS analysis of PVA coated by 3.85 mg/mL PDA; IR spectra of PVA@BSA with different mass ratios; TGA of PVA@BSA (2:1) and (1:3); long-term stability of PDA@PVA@BSA fibers; cytotoxicity study of PDA@PVA@BSA fibers; CHN elemental analysis of PVA@BSA and PDA@PVA@BSA; BSA release from PDA@PVA@BSA with different coating times and with different concentrations; CHN elemental analysis results of PVA@BSA coated by PDA for different times and with different concentrations (PDF)
