# Supplementary material for: Polydopamine-Coated Polymer Nanofibers for In Situ Protein Loading and Controlled Release
Source: ACS Omega. 2024 Mar 13;9(12):14465–74. doi: 10.1021/acsomega.4c00263 (PMC10976389; doi:10.1021/acsomega.4c00263)
Supplement: Supplementary file 1 — ao4c00263_si_001.pdf [file ao4c00263_si_001.pdf]

Supporting Information

Polydopamine-Coated Polymer Nanofibers for in Situ Protein Loading and Controlled Release

Meina Zhang[1], Romy A. Dop[1,2], Haifei Zhang[1]*

[1] Department of Chemistry, University of Liverpool, Crown Street, Liverpool L69 7ZD, UK.

[2] Department of Clinical Infection, Microbiology and Immunology, Institute of Infection, Veterinary and Ecological Sciences, University of Liverpool, Liverpool L69 7ZD, UK

*Corresponding author email: zhanghf@liverpool.ac.uk

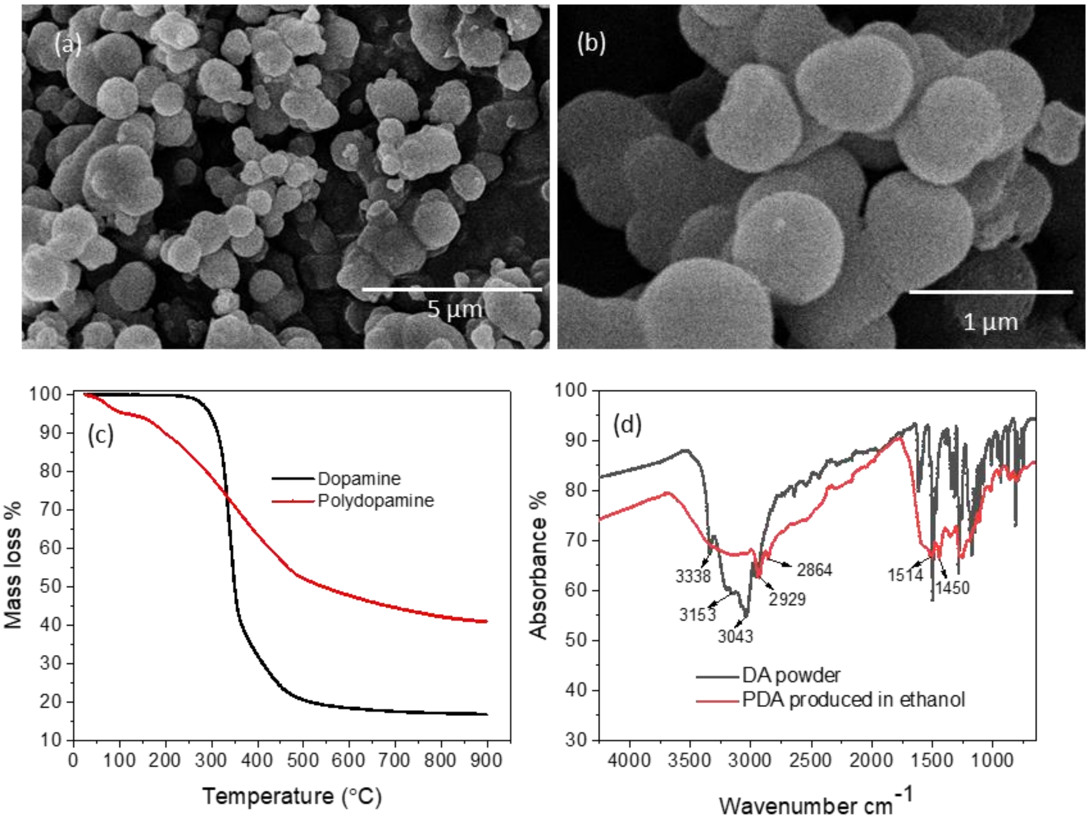

Figure S1. (a) and (b) Microstructure of PDA nanoparticles at different magnifications. (c) and (d) TGA profiles and FTIR spectra of DA and PDA.

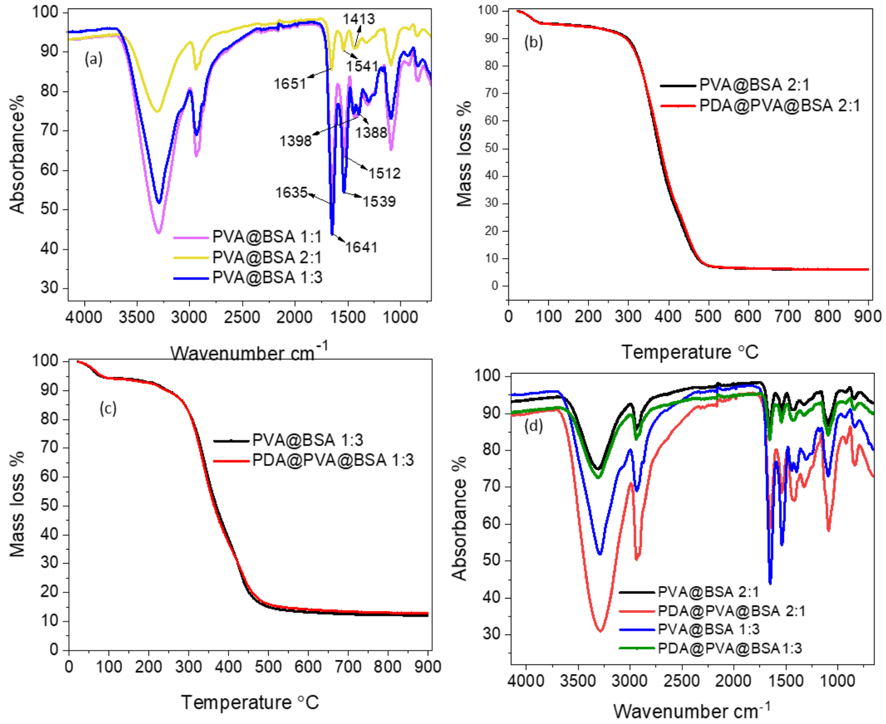

Figure S2. (a) FTIR spectra PVA@BSA with different mass ratios. (b) and (c) Mass loss curves of PVA@BSA (2:1) and (1:3). (d) IR spectra of PVA@BSA (1:1) and (2:1) coated by PDA.

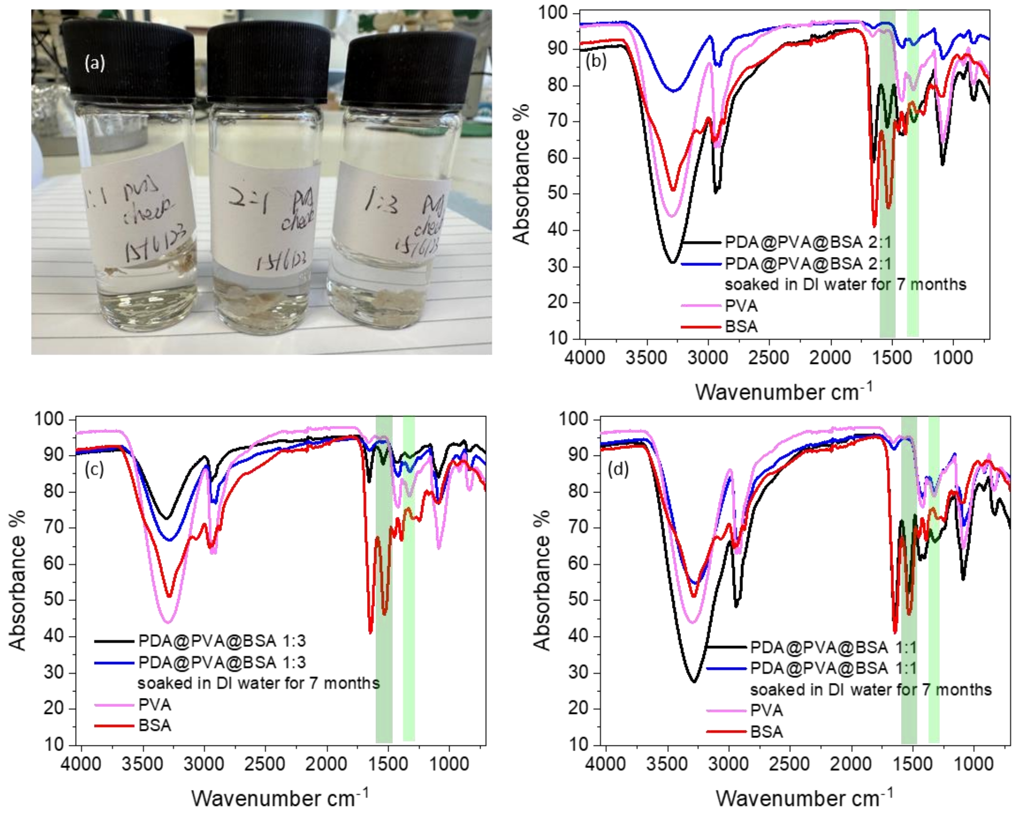

Figure S3. (a) Photograph of PDA@PVA@BSA (1:1, 2:1, and 1:3) in water. (b), (c) and (d) FTIR spectra of soaked PDA@PVA@BSA (1:1, 2:1, and 1:3) in water for 7 months.

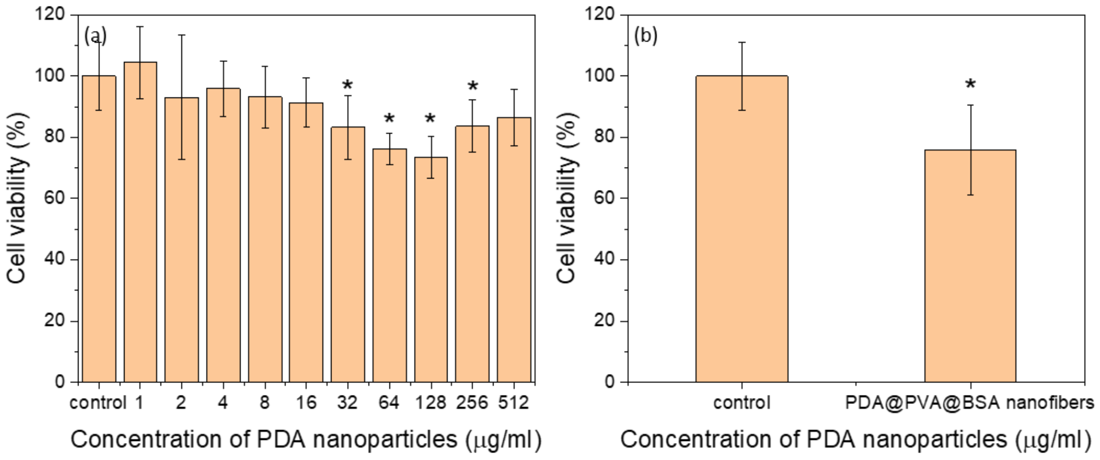

Figure S4. Cell viability (%) of A549 cells (a) after PDA nanoparticle treatment with different concentrations and (b) after PDA@PVA@BSA treatment (2:1). * $p < 0.05$ relative to control of cells in absence of nanoparticles.

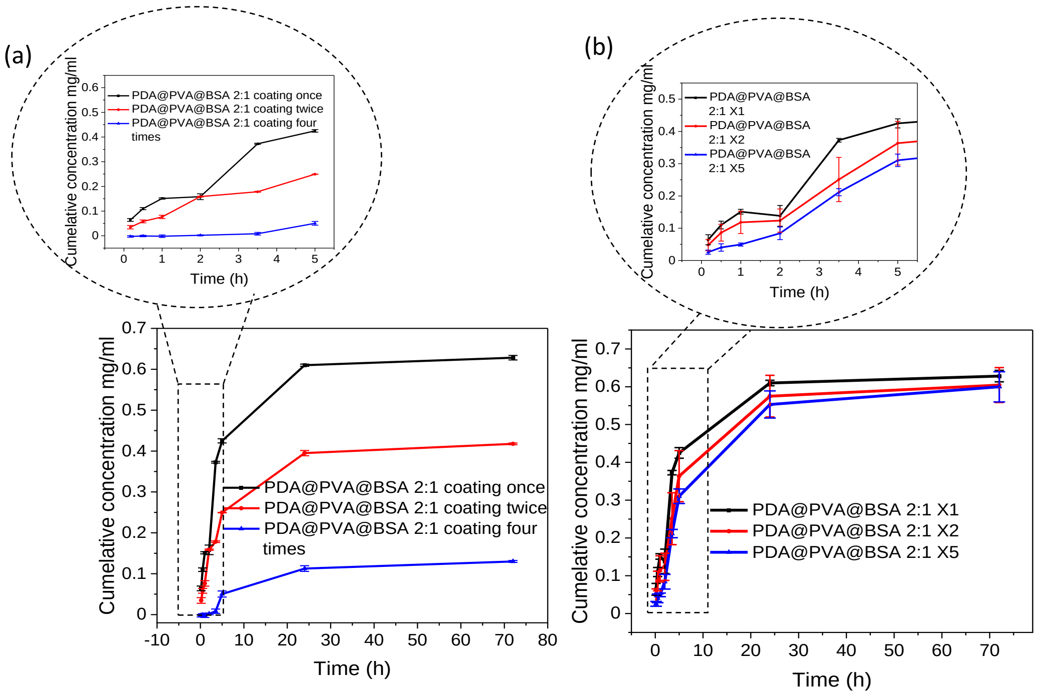

Figure S5. BSA release concentration of BSA from PDA@PVA@BSA with different coating times (a) and with different concentrations (b).

Table S1 CHN elemental analysis results of PVA fibers, PDA nanoparticles and PVA coated by PDA in different conditions

	C	H	N
PVA fibers	50.68%	8.64%	0
PDA nanoparticles	58.98%	5.24%	9.63%
PVA coated by 3.85 mg/ml PDA	52.64%	8.69%	0.33%
PVA coated by 1.54 mg/ml PDA	52.21%	8.89%	0
PVA coated by 0.77 mg/ml PDA (24 ml DA-ethanol solution for 4 h at room temperature and 17 h in freezer)	51.98%	8.84%	0
PVA coated by PDA in 3 ml ethanol	51.65%	8.84%	0
PVA coated by PDA in 7 ml ethanol	51.65%	8.84%	0
PVA coated by PDA in Freezer for 21 h	51.43%	8.38%	0
PVA coated by PDA at room temperature for 21 h	51.61%	8.51%	0

Table S2 EDS results of PDA@PVA 3.85 mg/ml

Element	Mass%	Atom%
C K	72.74%	66.77%
N K	0.61%	0.64%
O K	26.65%	36.59%

Table S3. CHN elemental analysis results of PVA@BSA and PDA@PVA@BSA

	C	H	N
BSA	48.43%	6.92%	13.86%
PVA fibers	50.68%	8.64%	0
PDA	58.98%	5.24%	9.63%
PVA@BSA (1:1)	49.49%	7.76%	6.29%
PDA@PVA@BSA (1:1)	50.83%	7.82%	7.01%
GA@PVA@BSA (1:1)	51.66%	7.76%	6.21%
PVA@BSA (2:1)	50.05%	8.14%	4.18%
PDA@PVA@BSA (2:1)	50.87%	8.05%	4.53%
PVA@BSA (1:3)	49.25%	7.45%	9.87%
PDA@PVA@BSA (1:3)	49.82%	7.28%	10.73%

Table S4. CHN elemental analysis results of PVA@BSA coated by PDA for different times and with different concentration

	C	H	N
PDA@PVA@BSA (2:1) once and X1	50.87%	8.05%	4.53%
PDA@PVA@BSA (2:1) twice	51.08%	7.96%	4.68%
PDA@PVA@BSA (2:1) four times	51.56%	7.95%	4.93%
PVA@BSA (2:1) X2	49.36%	7.96%	4.18%
PDA@PVA@BSA (2:1) X2	50.03%	7.99%	4.47%
PVA@BSA (2:1) X5	49.41%	7.88%	3.86%
PDA@PVA@BSA (2:1) X5	49.77%	8.11%	4.29%
